# Immune characteristics of olfactory ensheathing cells and repair of nerve injury

**DOI:** 10.3389/fimmu.2025.1571573

**Published:** 2025-05-15

**Authors:** Ding-yi Chen, Wen-jun Zhang, Cheng Zuo, Yong-sheng Xu, Liu-xiang Fu

**Affiliations:** ^1^ Department of Emergency Medicine, The Second Affiliated Hospital, Jiangxi Medical College, Nanchang University, Nanchang, Jiangxi, China; ^2^ Department of Rehabilitation Medicine, The Second Affiliated Hospital, Jiangxi Medical College, Nanchang University, Nanchang, Jiangxi, China; ^3^ The Second Affiliated Hospital, Jiangxi Medical College, Nanchang University, Nanchang, Jiangxi, China

**Keywords:** OECS, immune regulation, anti-inflammation, nerve injury repair, regeneration

## Abstract

The process of nerve injury is accompanied by the change of inflammatory microenvironment, which is not conducive to axonal regeneration and hinders the repair of injured nerve. Therefore, looking for a way to improve the inflammatory attack and immune state around the injured nerve is beneficial to the progress of nerve injury repair. In recent years, cell transplantation strategy has played a foreground role in the repair of nerve injury. Olfactory ensheathing cells (OECs) are a special kind of glial cells, which have the characteristics of continuous renewal and survival, antigenic characteristics, variability and promoting the repair of nerve injury. OECs have been recognized in different injury models, including clinical trials, which has become a dominant cell in cell replacement therapy. An important feature of OECs lies in their anti-inflammatory and immunomodulatory functions. They are transplanted into the host to improve the catastrophic inflammatory microenvironment caused by injured nerves, thus promoting the repair and regeneration of injured nerves. The transplantation of OECs into the host can provide good groundwork and support for the repair and regeneration of nerve injury by regulating the activity and infiltration of immune cells, the secretion of inflammatory cytokines and phagocytosis. Therefore, this paper discusses the anti-inflammatory and immunomodulatory mechanisms of OECs transplantation in the repair of nerve injury and the functional role of OECs as an ideal substitute in the treatment of nerve injury.

## 1 Introduction

Nerve injury is a common problem at present, which can be induced by entrapment, trauma, ischemia, inflammation and immunity, which can lead to a variety of defects, including loss of sensation, loss of movement and chronic pain. These not only bring devastating blows and psychosomatic injuries to patients, but also bring huge economic burden to the family and society (1–3). The ability of injury and regeneration of central nervous system in adult mammals is limited. Although the ability of peripheral nerve regeneration is stronger than that of central nervous system, repair and regeneration cannot be completed after injury. After nerve injury, ischemia, swelling, and bleeding may occur, accompanied by a series of adverse factors, including activation of inflammatory cells, hindering nerve regeneration and functional recovery (4, 5). Although inflammation is a protective mechanism after nerve injury, excessive inflammation can lead to widespread damage and exacerbate disease progression. In these processes, what is more important is that the activity of immune cells around the nerve injury increases and penetrates, thus releasing a variety of inflammatory cytokines and forming an inflammatory microenvironment that is not conducive to the repair of the damaged nerve. During the progression of nerve injury, these inflammatory cells, such as microglia and macrophages, can be activated. These activated cells can migrate and gather at the injury site and by changing their phenotypic changes (pro-inflammatory phenotype), release a variety of pro-inflammatory cytokines and chemokines, induce neuroinflammation, produce neurotoxic effects, and hinder neuron survival and axon regeneration (6, 7).

Central nervous system damage can trigger microglia activation within minutes, and then immune cells in the blood are attracted to chemokines and infiltrate under the influence of the destruction of the blood-brain barrier. Blood-borne monocytes differentiate into phagocytic macrophages, which, together with microglia, constitute innate immunity and are responsible for clearing debris and providing a source of nutritional and anti-inflammatory factors to promote tissue repair, but they also release inflammatory cytokines to promote secondary damage (8, 9). During the secondary injury stage, it can cause vascular damage, ischemia and hypoxia, cell necrosis, neuroinflammation, etc., which can lead to aggravation of tissue damage. Neuroinflammation is an important aspect of the pathological process, which usually involves the aggregation of resident microglia, astrocytes, macrophages and neutrophils. At the beginning of inflammation, the levels of cytokines such as macrophage inflammatory protein-1a (MIP-1a), IL-1, IL-6 and tumor necrosis factor (TNF-a) are u-regulated at the injury site, thereby triggering a cascade amplification reaction through receptor action, prolonging and amplifying nerve damage, causing a vicious cycle and seriously damaging the microenvironment at the nerve injury site (10, 11). In this catastrophic environment, nerve regeneration can be challenging. Therefore, this change in the inflammatory microenvironment during nerve injury increases or hinders the nerve repair process, greatly hindering axon regeneration, nerve bridging, and myelin formation (12–14). Different studies have confirmed that inflammation plays an important role in tissue repair and functional recovery (15). Therefore, it is of great significance for the repair of nerve injury and functional recovery to explore foreground methods to improve the inflammatory microenvironment of nerve injury.

As people continue to explore in this field, in recent years, cell transplantation technology has entered people’s field of vision. The researchers can repair the injured nerve by obtaining functional cells and transplanting them into the host (16, 17). For example, Schwann cells (SCs) implantation combined with electroacupuncture can promote axonal regeneration and myelin regeneration of corticospinal tract after spinal cord injury by up-regulating the expression of neuRegin1 type III in SCs and their downstream signal media (18). OECs are a special kind of glial cells, which can survive and renew for life in the central and peripheral nervous system. The primary olfactory system is characterized by its ability to produce new neurons in adult animals. This special ability is due in part to the presence of OECs, which creates a favorable microenvironment for neurogenesis. OECs promote the continuous regeneration of neurons in the olfactory system (19). OECs can be directly, quickly and effectively reprogrammed into neurons via the single transcription factor Neurogenin 2 (NGN2). These inducing cells exhibit typical neuronal morphology, express multiple neuron-specific markers, generate action potentials, and form functional synapses. Genome-wide RNA sequencing analysis shows that OECs transcriptome profiles can be effectively reprogrammed into neuronal lineages (20). Moreover, OECs are rich in sources and can be derived from olfactory bulb tissue and olfactory mucosa. Primary OECs are easier to obtain from embryonic animals and early postnatal animals, but it is more difficult to obtain purified OECs due to the large number of non-OECs in the embryonic and early postnatal olfactory bulbs and olfactory mucosa. In contrast, OECs can be easily purified and cultured from adult olfactory bulbs and olfactory mucosa to obtain highly viable and highly pure OECs (19). Because of these capabilities, people are exploring the use of OECs in the treatment of nerve injury repair (such as spinal cord injuries) and making significant progress. The mechanisms of OECs transplantation in promoting the repair and functional recovery of injured nerve include promoting axonal regeneration and myelination, secreting a variety of neurotrophic factors and neuroprotection (19, 21, 22). In addition, another important mechanism of OECs promoting the repair of nerve injury can improve this inflammatory microenvironment and exert the characteristics of anti-inflammation and immune regulation. OECs can regulate the activity of immune cells, inhibit the release of inflammatory mediators, and provide a good local microenvironment for axonal regeneration and neuronal regeneration (23, 24). After OECs transplantation, the expression of interleukin-1 receptor antagonist (IL-1ra) in spinal cord tissue was up-regulated, and the expression of many chemokines, including proinflammatory chemokine IL-1α and IL-1β, was down-regulated, which played an anti-inflammatory role (25). OECs can also significantly inhibit the activity of microglia stimulated by lipopolysaccharide and reduce neuroinflammatory response (25). In recent years, OECs have been used in clinical trials, which has confirmed the feasibility and safety of OECs transplantation. Therefore, we explored the anti-inflammatory and immunomodulatory effects of OECs in nerve injury repair, so as to provide a favorable microenvironment for nerve regeneration.

## 2 Origin of OECs

OECs are glial cells located in the primary olfactory nervous system, which originate from the neural crest. and exist in the nerve fiber layer of the olfactory nerve, olfactory mucosa and olfactory bulb of the system (26). Neural crest cells shed from the dorsal nerve canal through epidermal-mesenchymal transformation (EMT), migrate along the olfactory nerve to the lamina propria of the olfactory mucosa, and pass through the epithelium to the basal margin. Then, neural crest cells-derived OECs can still extend along the olfactory nerve to the telencephalon of the central nervous system, wrapped around the olfactory axon bundle. The olfactory axon of the olfactory nerve ends close to the olfactory bulb, and finally reaches the olfactory nerve layer (ONL) of the olfactory bulb and matures here (22, 27). When the axon grows through the basal layer of the epithelium, it passes through the basement membrane and enters the lamina propria, and combines to form an axonal bundle, which is surrounded by OECs (22). The primary olfactory nervous system is unique in that it can constantly regenerate, even after an injury, as long as the deeper olfactory bulb remains intact. The olfactory bulb tissue contains abundant OECs, which can grow and regenerate olfactory neurons and maintain the function of the olfactory system. This also shows the important role of OECs in the development of the primary olfactory nervous system (28, 29). OECs have unique growth and migration promoting properties, as well as biological functions directly related to neuron survival and axon extension. OECs in the olfactory nerve promote axon bundle formation, while OECs from the olfactory bulb nerve fiber layer mediate unbundling, sequencing, and rebundling of axons and guide axons to their appropriate bulb targets (30, 31). OECs wrap bundles of olfactory axons around the olfactory nerves and in the olfactory nerve layer of the olfactory bulb, and these primary olfactory axon bundles surrounded by OECs support the regeneration of primary olfactory axons from the peripheral to the central nervous system and from the olfactory epithelium to the bulb (32, 33). The Sox10 transcription factor exists in the olfactory nerve and can directly regulate the differentiation and expression of OECs (31). When OECs migrate to the basal edge of the development site, OECs are formed. There are many OECs around the olfactory bulb and in the ONL. ONL is divided into inner layer and outer layer, the inner layer cannot express p75NTR, and the outer layer can express p75NTR. OECs play a key role in neurite growth and olfactory nerve axis function, and can be completely regenerated to maintain sensory function even after extensive injury (31).

OECs culture are a source of cells needed for nerve injury treatment. This is due to its advantages, such as high migration ability, easy source (can be derived from nasal olfactory mucosa or olfactory bulb tissue), simple *in vitro* culture, and non-tumorigenicity (34). At present, OECs from autologous or allogeneic origin can be obtained through *in vitro* culture systems. In basic research, olfactory bulb tissue or olfactory mucosa (the lamina propria of the olfactory mucosa consists of OECs covering olfactory nerve fibers) can be taken from animals (such as dogs, rats and mice) and can be obtained by culture *in vitro*. These cultured OECs are further identified by using the OECs-specific marker p75NTR (35–37). The human nasal mucosa is located in the middle and superior turbinate and nasal septum of the nasal cavity, while the olfactory mucosa plays an important role in smell and is the only nerve tissue exposed to the external environment. This feature allows people to easily access the olfactory mucosa (34). It is worth mentioning that the production of OECs in young patients is relatively high. Higher yields of OECs can be obtained from specimens collected from the tail of the superior turbinate, while cell culture is poor in patients with severe mucosal disease or elderly patients (38–41). This also means that the culture rich in human OECs and the cells with high viability and yield can be obtained by selecting the olfactory mucosa of appropriate age and location. Although the *in vitro* culture method of OECs is relatively mature and stable, for clinical trials, the source of cells and the ethical issues are a major challenge hindering clinical development.

## 3 Heterogeneity and antigenicity of OECs

Another important feature of OECs is their antigenicity, morphological and functional heterogeneity. In the early stage, it is shown that there are different antigenic OECs subsets in the olfactory system, which is closely related to their location and development. Now studies have shown that OECs in the olfactory nerve and olfactory bulb express a series of antigens, which can be detected and identified by various cellular and molecular techniques (23, 24). OECs in the lamina propria can strongly express S100 β and weakly express glial fibrillary acidic protein (GFAP) and nerve cell adhesion molecules. OECs in the olfactory nerve layer express low neurotrophic receptors p75, neural cell adhesion molecule (NCAM) and GFAP, while the OECs in the olfactory bulb do not express these molecules (23). Due to the heterogeneity and anatomical location of OECs, there are differences in the expression of some antigenic factors. For example, neuropeptide Y is expressed in OECs during the development of olfactory nerve layer, but not in OECs of olfactory nerve layer and peripheral olfactory system in adulthood, which is closely related to the heterogeneity of OECs (23, 24).

There are also differences in morphology of OECs, which reflects the functional role of OECs in the repair of nerve injury. OECs can be similar to SCs and astrocytes and undergo morphological changes due to external and internal environmental changes (42). Although OECs express many molecular and cellular characteristics of SCs and astrocytes, there are significant differences in molecular expression between OECs and these two kinds of cells (43). OECs are mainly protuberant under pro-inflammatory conditions and flat under anti-inflammatory conditions (43). Compared with the flat OECs under anti-inflammatory conditions, the OECs carried by processes showed higher cell metabolic activity and higher mobility, and significantly promoted the growth and extension of axons under anti-inflammatory conditions. Studies have shown that transcriptional coactivator related protein (YAP) downstream of ROCK pathway mediates the morphological transformation of OECs and enhances their ability to promote axonal growth by up-regulating the expression of L1 cell adhesion molecule (L1-CAM) (43). Inhibitors of RhoA pathway, toxin B, C3, Ymur27632 or overexpression of dN-RhoA blocked the morphological changes of OECs from processes to flattening induced by serum, while lysophosphatidic acid activated RhoA pathway promoted the morphological changes of OECs from processes to flattening (44). These studies have revealed the plasticity and heterogeneity of OECs.

In addition, OECs derived from olfactory mucosa (OM) can regulate inflammatory process and the formation of extracellular matrix, but have poor ability of regeneration, while OECs derived from olfactory bulb (OB) can promote functional recovery by inducing targeted axonal regeneration (19). OM-OECs overexpress genes characteristic of wound healing and extracellular matrix regulation. In contrast, the genetic profile of OB-OECs has been shown to play an important role in nervous system development (45). OECs were extracted from OB and OM, and gene and protein expression profiles of cells were compared using transcriptomics and non-quantitative proteomic techniques. The results showed that both OB-OECs and OM-OECs highly express genes and proteins that regulate cell growth, reproduction, cell death, and vascular endothelial cell regeneration. Differentially expressed genes and proteins in OB-OECs play a key role in regulating nerve regeneration and axon regeneration and extension, nerve impulse transmission and response to axon injury. The differentially expressed genes and proteins in OM-OECs are mainly involved in the positive regulation of inflammatory responses, defense responses, cytokine binding, cell migration and wound healing (46). There are 2164 differentially expressed genes in OB-OECs in patients with Parkinson’s disease, of which 1090 are up-regulated and 1074 are down-regulated. The most significantly expressed genes include down-regulated genes CHRNA3, SNCG, OR7D2 and up-regulated genes TOP2A, RM2 and IGLC2. Important pathways rich in down-regulated genes include neurodegeneration, oxidative phosphorylation, and olfactory conduction. Pathways rich in upregulated genes include several pathways involved in neuroinflammation, immune and inflammatory responses, such as NF-kB, Jak-STAT, toll-like signaling pathways, and pathways involving pro-inflammatory mediators (47). These findings suggest that differentially expressed genes and proteins can explain why OB-OECs and OM-OECs exhibit different therapeutic effects.

## 4 Phagocytosis of OECs in the repair of nerve injury

Nervous system injury is characterized by neuronal degeneration and death, persistent cell and apoptotic fragments, as well as inflammatory response and immune damage, which create an adverse environment for nerve survival and axonal regeneration (23). Therefore, in the process of nerve repair, timely cleaning/removal of denatured/apoptotic cells and fragments and inhibition of inflammatory response are of great significance for the repair of injured nerve. Glial cells play an important role in the maintenance and stability of nervous system function. Glial cells can respond quickly to nerve injury by removing fragments from the injured site and providing necessary growth factors and structural support, all of which provide support for neuronal regeneration (48, 49). During the development of the primary olfactory system, the axons are inaccurately located, and the axons inappropriately project to the target layer or over-project to the deep layer of the olfactory bulb. In addition, there is a large number of apoptosis of primary olfactory neurons during embryonic and postnatal development, and axons of degenerated neurons need to be removed (50). The olfactory nerve is constantly renewed throughout life, and the OECs maintain the stability of the olfactory system and constantly stimulate nerve regeneration (48). The phagocytosis of OECs to clear neuronal fragments has been shown to contribute to the growth of neurons. OECs can exert phagocytosis by secreting immunomodulatory molecules, thus maintaining microenvironmental homeostasis and supporting neuronal survival and axonal growth (32, 51). In fact, OECs can remove axonal fragments and bacteria through their morphological and phenotypic changes, including cytoskeleton hypertrophy and rearrangement, transition from resting state to phagocytic phenotype, and protect olfactory nerves from microbial infection (24, 51). Studies have shown that the phagocytosis of primary olfactory axon fragments by OECs occurs at 14.5 days of the embryo, and the phagocytosis of axonal fragments continues to enter postnatal animals during the period of widespread mispositioning of olfactory axons (52).

OECs respond to phagocytosis of bacteria, which may be essential for responding to microbes invading the central nervous system through the peripheral nerve. SCs can help remove fragments by attracting macrophages to the injured site during the repair of peripheral nerve injury (52). But unlike SCs, OECs in the olfactory system do not seem to attract macrophages (52). Studies have shown that OECs have higher phagocytosis and transport capacity than SCs and produce lower amounts of proinflammatory cytokines (50). Macrophage migration inhibitory factor (MIF) is an important innate immune regulator, which plays a key role in many functions such as nerve regeneration and response to pathogens, except the primary olfactory nervous system. MIF participates in the fact that a large number of macrophages are missing in the olfactory nerve bundle and strongly stimulates the phagocytosis of OECs (53). It has been found that macrophages often appear near OECs after nerve injury, but they only play a small role in clearing axonal fragments (33). These mean that OECs are the primary phagocytes of the primary olfactory nerve from the early stage of embryonic development. Interestingly, the conditioned medium of macrophages co-incubated with platelet-derived growth factor (PDGF) can promote the phagocytosis of OECs and regulate the expression of myelin sheath genes related to nerve repair in OECs under inflammatory conditions (54).

OECs express growth-promoting adhesion molecules and extracellular matrix molecules, and bind closely to olfactory axons in space, which is consistent with the involvement of OECs in promoting and guiding olfactory axon growth (42). OECs secrete anti-inflammatory cytokine transforming growth factor β1 (TGF-β1) in the process of phagocytosis and removal of neuronal fragments. TGF-β1 enhances phagocytosis of OECs by regulating integrin/MFG-E8 signal pathway (55). TGF-β1 makes OECs move to flat direction and increase cell area, which may also participate in the enhancement of phagocytosis of OECs, promote OECs to clear neuronal fragments and increase neuronal survival rate (55). In the model of primary cultured spinal cord neurons *in vitro*, it was found that OECs could phagocytize a large number of degenerated neuronal fragments, which further showed that OECs increased the length of neuronal axons and enriched the number of neurons with axonal branches (32). In addition, the phagocytic activity of OECs does not affect the production of bioactive molecules, which provides a favorable environment for the survival of neurons (32). Through the co-culture model *in vitro*, it was found that the combination of lipopolysaccharide and curcumin could significantly enhance the activity of OECs, up-regulate the expression of chemokine (C-X-C motif) ligand 1, C-X-C motif ligand 2, TNF-α and Toll-like receptor 4 in OECs, enhance the phagocytosis of OECs and greatly promote the growth of neurons (56). These studies have revealed the key role of OECs in maintaining and stabilizing the function of the nervous system, creating an environment conducive to the growth of neurons by swallowing apoptotic and degenerative cells. Therefore, this functional characteristic of OECs may play an important role in the mechanism of nerve injury repair. However, more studies need to reveal the phagocytic ability in the repair of nerve injury.

## 5 Neuroinflammation mediated by immune regulation of OECs

Neuroinflammation mediated by immune cell activation and infiltration and the release of cytokines play an important role in nerve injury (49, 57, 58). The polarization of microglia/macrophages shows pro-or anti-inflammatory phenotypes, which can change the local microenvironment, process and severity of injury. These processes include the release of pro-inflammatory and anti-inflammatory cytokines, such as IL-1 α, IL-6, IL-8, IL-13, TNF-a and chemokine granulocyte colony stimulating factor and granulocyte macrophage colony stimulating factor (59, 60). Resident microglia near the injury site are activated, and neutrophils, macrophages, lymphocytes and natural killer cells are recruited to gather and infiltrate to the injury site, causing inflammatory damage by releasing cytokines such as free radicals, reactive oxygen species (ROS) and nitric oxide (NO) (23, 24). Therefore, it is necessary to stimulate nerve regeneration and find effective ways to regulate the transition of microglia/macrophages to neuroprotective phenotypes.

OECs have been shown to be beneficial to nerve injury by regulating immune cell activity and nerve inflammation, produce anti-inflammatory effects, support neuronal survival and promote angiogenesis (61, 62). OECs transplantation can improve the immune microenvironment at the transplant site by inhibiting the activity of immune cells, including macrophages and microglia, and the release of inflammatory cytokines, and create a favorable microenvironment for nerve regeneration (63). OECs can delay the activation of microglia/macrophages and reduce the peak of multiphasic inflammation and immune response in a time-dependent manner, resulting in neuroprotection and prevention of further inflammatory injury (64). In addition, studies have shown that OECs-derived exosomes (OECs-Exo) can inhibit the polarization of proinflammatory macrophages/microglia and increase the number of anti-inflammatory cells after spinal cord injury (64). OECs-Exo can be phagocytized by microglia and partially reverse endotoxin-induced pro-inflammatory polarization by inhibiting NF–κB and c-jun signal pathways *in vitro* (61). OECs-Exo can inhibit the changes of pro-inflammatory phenotype of macrophages/microglia, reduce neuronal death and protect neuronal survival and axons (61). OECs-Exo enriched in hsa-miRNA-6780-5p reduce polyglutamine (PolyQ) expression and increase autophagy levels, and inhibit Spinocerebellar Ataxia type 3 (SCA3) expression (65). Moreover, OECs prevent TNF-a induced cell death in neurons, in part through exosome-derived brain-derived neurotrophic factor (BDNF) (66). OECs overexpressed by nuclear receptor-associated factor 1 (Nurr1) and neuron 2 (Ngn2) increase the vitality of PC12 cells, inhibit oxidative stress and cell death, and show significant neuroprotective, antioxidant and anti-cell death effects (67). Aβ (25-35) triggers oxidative damage mediated by ROS, changes the structure and function of mitochondria, and leads to the activation of the intrinsic cell death pathway in mitochondria. Significant changes in cyclin D1 intensity and increases in total ROS levels are particularly observed in OECs exposed to toxic fragments of Aβ (25-35) (68).

**Figure 1:**
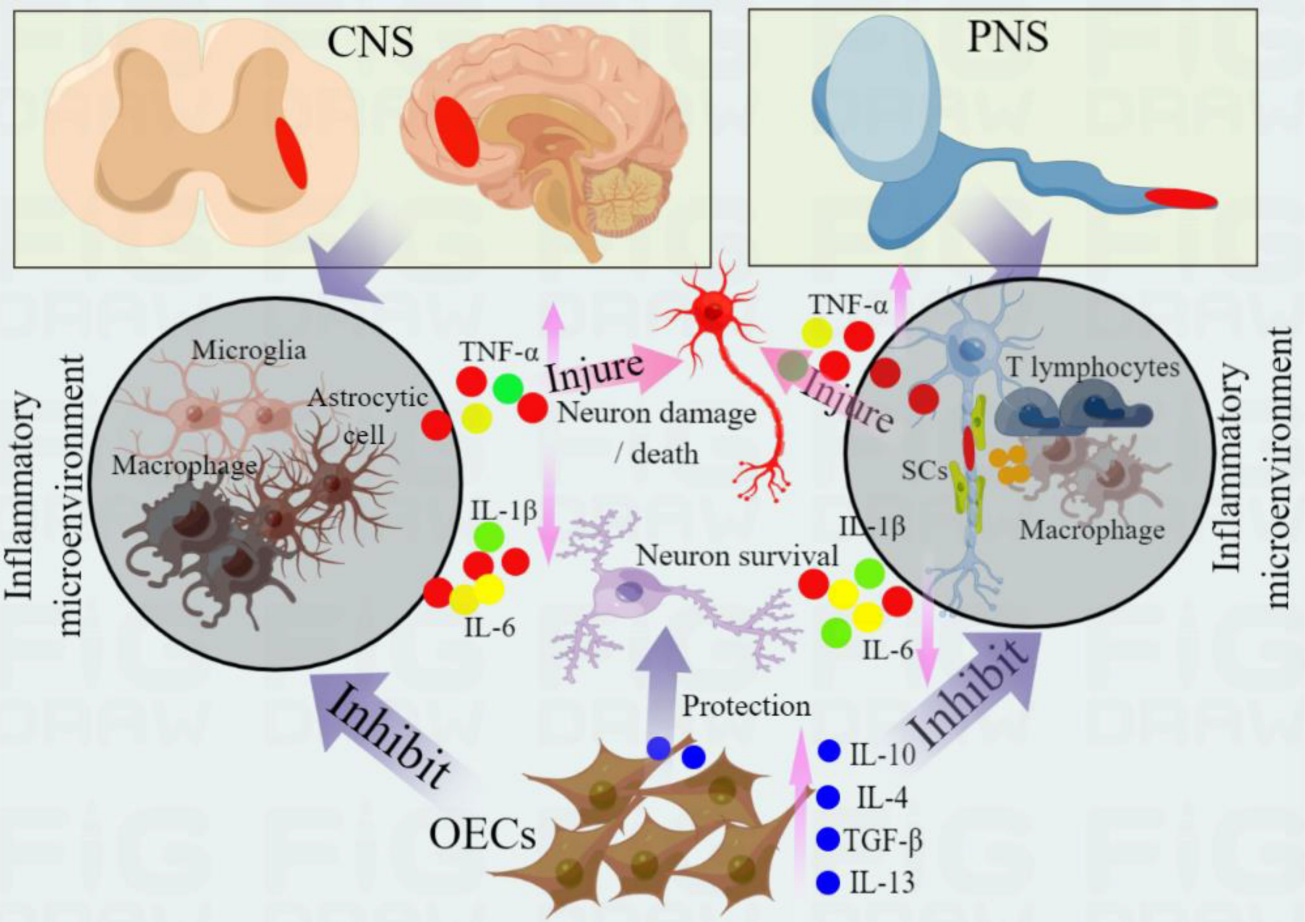
Immunomodulatory and anti-inflammatory effects of OECs in nerve repair. Central nervous system injury (such as spinal cord injury) can induce the activation and infiltration of immune cells (microglia, macrophages and astrocytes), while peripheral nerve injury (PNS) induces the activation of SCs, and activated SCs induce the migration and infiltration of inflammatory cells (such as macrophages and T lymphocytes) by releasing chemokines. These immune cells activation can release pro-inflammatory cytokines (such as IL-1β, IL-6 and TNF-α), further aggravate secondary injury, promote neuronal injury and apoptosis, and hinder nerve regeneration. OECs can regulate the activity of immune cells, change their polarization, promote the transformation of anti-inflammatory phenotype and reduce the release of proinflammatory cytokines. In addition, OECs express a variety of cytokines, which can improve the inflammatory environment, protect injured nerves and promote nerve regeneration and repair by secreting anti-inflammatory cytokines (such as IL-10, IL-4 and TGF- β). These anti-inflammatory cytokines produced by OECs can further inhibit the level of pro-inflammatory cytokines and protect the survival of neurons.

OECs regulate the polarization of microglia from pro-inflammatory phenotype (M1) to anti-inflammatory phenotype (M2) through APOE/TREM2/NF-κB pathway, which effectively reduces the malignant inflammatory response and promotes the functional recovery after spinal cord injury in rats (69). Further studies have shown that curcumin-treated OECs can effectively promote nerve survival and axonal growth. The transplanted OECs can improve the neurological prognosis of spinal cord injury in SD male rats. Its curative effect is largely attributed to the anti-inflammatory activity of OECs by regulating the polarization of microglia from M1 phenotype to M2 phenotype (70). Intravenous transplantation of OECs after spinal cord injury regulates microglia activation in the brain and spinal cord of SD rats by upregulating REV-ERBα (71). Moreover, IL-4 plays a leading role in triggering the phenotype of M1 to M2 microglia, significantly reducing the levels of M1 markers IL-1β, IL-6, TNF-α and inducible nitric oxide synthase, while increasing M2 markers Arg-1, TNF-β, IL-10 and CD206. This process coordinates the microglial polarization event regulated by IL-4 with the crosstalk between JAK1/STAT1/3/6 signal and NF-κB/SOCS1/3 signal (70). Studies have shown that curcumin activation of transplanted OECs can promote nerve regeneration and functional recovery after spinal cord injury in rats, and its mechanism is closely related to the production of neurotrophic and anti-inflammatory factors by OECs, reduction of pro-inflammatory cytokines, and significant reduction of cavity and glial scar formation (72) (**Figure 1**). These studies have revealed the immunomodulatory function of OECs in the process of nerve injury and regeneration, which can reduce nerve inflammation and promote regeneration by changing the activity of immune cells.

## 6 OECs produce cytokines conducive to the repair of nerve injury

The process of nerve regeneration is accompanied by the production of a variety of cytokines, and some inflammatory cytokines and chemokine-mediated inflammatory cascades aggravate nerve injury (73, 74). Therefore, improving this change, reducing the release of pro-inflammatory cytokines and increasing the production of anti-inflammatory cytokines are beneficial to nerve regeneration and functional recovery. OECs can regulate the injured area and promote the regeneration of injured nerve by producing growth factors and cytokines. OECs can produce anti-inflammatory cytokines, such as IL-4, IL-10, TGF-β and IL-13, which prevent cell degeneration or death by regulating the production of inducible nitric oxide synthase and nitric oxide under the stimulation of lipopolysaccharide/interferon-γ (64). These anti-inflammatory cytokines inhibit the release of pro-inflammatory cytokines, including TNF-α, IL-1β, IL-2 and IL-6. Most anti-inflammatory factors (IL-4, IL-10, IL-13 and TGF-β) derived from OECs are involved in regulating cell survival, proliferation and migration, thus promoting nerve regeneration after nerve injury (51, 75). OECs not only have strong innate immune regulation, but also can remove cell fragments under the mediation of cytokines (IL-10 and TGF-β) (51). There is also the expression of phosphatidyl serine (PS) receptor in OECs, which enables OECs to recognize apoptotic OECs and phagocytize apoptotic fragments by binding to phosphatidylserine (51). OECs express p75, S100β, and GFAP, which are characteristic markers of globular OECs (activated state). OECs also express a group of unique developmental important proteins-CD44, β1 integrin, P200, Notch3, NG2, vascular endothelial growth factor (VEGF), pituitary adenylate cyclase-activating polypeptide (PACAP) and CREB binding protein (CBP/p300), promote and facilitate nerve regeneration in the adult nervous system according to environmental stimuli and changes (76). OECs attenuate the pro-inflammatory response of human brain microvascular endothelial cells under hypoxia, reduce the HIF-1α/VEGFA signal activation pathway, reduce IL-8 levels, and produce anti-inflammatory effects (77).

Astrocytes play a key role in central nervous system inflammation and nerve regeneration (53, 54). Activation of glial cells leads to the release of specific chemokines and pro-inflammatory cytokines, including IL-1, IL-6 and tumor necrosis factor. These cytokines prevent axonal regeneration by activating their respective cascade reactions, amplifying the inflammatory response, changing the microenvironment, and promoting cell death, thereby preventing axonal regeneration (78, 79). OECs can regulate the response of astrocytes and create an environment conducive to regeneration. Studies have shown that IL-10-mediated upregulation of Matrix metalloproteinase-13 (MMP-13) reduced the interaction between OECs and reactive astrocytes and inhibited glial scars (80). In addition, NF-κB is a key transcriptional regulator of inflammatory genes. Stimulation of p65 NF-κB translocation to the nucleus provides a basis for inflammatory activation of astrocytes (81, 82). Molecules released by OECs can inhibit the activation of NF-κB and play a neuroprotective role after central nervous system injury. Astrocytes cultured in OECs conditioned medium showed a decrease in nuclear translocation of NF-κB, which is a pro-inflammatory protein that induces neurotoxicity of astrocytes (83). Soluble factors released by OECs significantly inhibit NF-κB translocation in astrocytes induced by PMA/calcium carrier or microglial derived factor, which may be related to the expression of insulin-like growth factor-1 in OECs to significantly slow down NF-κB translocation in astrocytes (84). In addition, OECs can produce α B-crystallin (CryAB) through paracrine, which is an anti-inflammatory protein that coordinates the immune response between OECs and astrocytes (83). This suggests that CryAB and other factors secreted by OECs are potential drugs that can improve or even reverse the growth inhibitory environment created by neurotoxic reactive astrocytes after nervous system injury. Moreover, OECs significantly inhibit the transcription of proinflammatory cytokines and granulocyte-macrophage colony-stimulating factor in activated astrocytes (84).

OECs can regulate the microglia-astrocyte response by secreting anti-inflammatory cytokines such as IL-4, IL-10, IL-13 and TGF-β, thus down-regulating pro-inflammatory factors IL-1β, tumor necrosis factor and IL-6 (23). After OECs transplantation, the expression of interleukin-1 receptor antagonist (IL-1ra) in spinal cord tissue was up-regulated, while the expression of many chemokines, including proinflammatory chemokine IL-1 α and IL-1 β, was down-regulated (25). *In vitro* studies have confirmed that lipopolysaccharide stimulates OECs to secrete IL-1ra, while IL-1ra gene knockout significantly reduces its ability to regulate the activity of microglia, thus reducing neuroinflammation (25). In addition, OECs express chemokine and their homologous receptors, such as chemokine (CXC motif) ligand 1 (CXCL1), which may play a role in embryonic development or after transplantation of OECs at injured sites. CXCL12, CXCL4, and chemokine (CX3C motif) ligand 1 (CX3CL1) have been shown to play a key role in neuroinflammation as a signal factor for neutrophil and various leukocyte recruitment (23, 85). These studies reveal a fact that OECs can express and secrete a variety of anti-inflammatory cytokines and inhibit the inflammatory response after nerve injury, which is beneficial to the repair of injured nerve. However, the cytokines produced by OECs are still complex in improving the process of neuroinflammation, and more studies are needed to clarify this mechanism. If we can fully understand the anti-inflammatory function of OECs, making use of this feature can bring certain value to the repair of nerve injury and functional recovery.

## 7 Present situation and challenge of immune characteristics of OECs in clinical trials of nerve injury

Based on the reliable results of the basic research on the application of OECs in the repair of nerve injury. In recent years, researchers have begun to explore the rise from the basis to the clinical trial stage. Different studies have confirmed the feasibility and safety of clinical trials of OECs (86, 87). A clinical trial reported the therapeutic effect of OECs transplantation in 108 patients with chronic spinal cord injury. The results showed that 14 cases changed from American Spinal Injury Association (ASIA) A to ASIA B, 18 cases changed from ASIA A to ASIA C, 9 cases improved their walking ability or made them walk again with or without walking assistance, and 12 of 84 males improved their sexual function. 31 patients underwent MRI examination, and no pathological changes such as tumor, hemorrhage, swelling, cyst, nerve tissue destruction or infection were found in or around OECs transplant sites (88). Electromyography was performed in 31 patients, 29 cases were improved and 2 cases had no change. Paravertebral sensory evoked potential (PVSEP) test was performed in 31 patients, of which 28 cases were improved and the other 3 cases had no change. In addition, no deterioration or complications were found in all patients during the follow-up period (88). This clinical trial reveals that OECs treatment is safe, improves neurological function and improves quality of life in patients with fully chronic SCI. Another clinical trial reported that six patients with chronic complete spinal cord injury were treated with autologous OECs transplantation. After 24 months of follow-up, the standard neural classification scores of ASIA and International Society of Neurorepair Spinal Cord injury rating scale (IANR-SCIFRS) were significantly improved. No clinical complications occurred (89). This suggests that OECs transplantation appear to be clinically safe and may promote neurological recovery in SCI.

A recent case reported the effect of OECs transplantation in a patient who received OECs transplantation one year ago for complete traumatic spinal cord injury in the C6-C7 segment. Within a few days after cell therapy, the patient began to show clinical improvement. Six-year follow-up showed that his ASIA changed from ASIA-A to ASIA-C (90). The score of the International Society of Neurorepair Spinal Cord Injury Functional Rating Scale changed from 14 (prior to cell therapy) to 31+/-3 (six years after cell therapy). The main improvements in his daily life activities include eating, dressing and writing, standing and walking, urine control or urination. His sexual function returned to normal (90). Although this report reflects the beneficial effects of OECs transplantation, but more data are needed to support the reliability of the treatment effect. Other clinical trials have reported the transplantation of fetal OECs derived from olfactory bulb tissue via injection into the upper and lower ends of spinal cord injury sites in 300 patients (222 of whom had complete chronic SCI and 78 had incomplete chronic SCI). All patients were evaluated using ASIA criteria before and 2–8 weeks after transplantation. The partially-improved neurological functions assessed by the ASIA standard were indicated by the motor scores increasing from 39.1 +/- 20.6 to 45.9 +/- 20.3, the light touch scores from 51.7 +/- 24.9 to 63.4 +/- 23.0, and the pin prick scores from 53.0 +/- 24.2 to 65.3 +/- 22.7. There was no significant difference in the functional improvement of the motor, light touch, and pin brick when compared with the age, sex, duration after the injury, and the injury degrees and levels. The motor scores and light touch scores at the cervical level were higher than the scores at the thoracic level (91). This also suggests that OECs transplantation in the treatment of chronic spinal cord injury can quickly and partially improve neurological function. In addition, some studies have shown that co-transplantation of OECs with other cells (such as SCs and MSCs) into patients has good tolerance, which can improve the function and corresponding symptoms of patients after nerve injury (90, 92). For example, a phase one clinical trial evaluated the safety of cell transplantation by implanting autologous OEC and MSC into three patients with thoracic traumatic spinal cord injury through lumbar puncture. After 2 years follow-up, all adverse events and possible functional outcomes were recorded through general clinical examinations before and after surgery, magnetic resonance imaging (MRI), neurological assessment based on the SCI International Standards for Neural Classification, and functional assessment using the Spinal Cord Independence Measurement Version III (SCIM III). The results showed that no serious security issues were found. There was no change in MRI and no tumor tissue formation was found. ASIA improved from A to B in one of the participants. SCIM III evaluation also showed that the participant’s function had improved to some extent (92). The other two participants had negligible or no improvement in sensory scores, while there were no changes in AISA and SCIM III scores. No recovery from exercise was observed by any of the participants (92). This clinical trial did not produce any adverse results, which may indicate that a combination of autologous OEC and MSC is safe to treat SCI in humans. These studies have revealed the safety and feasibility of OECs in clinical trials. However, the immune and anti-inflammatory effects of OECs transplantation in patients have not been reported. There is no related literature to detect the changes of serum inflammatory and anti-inflammatory cytokines and the degree of local inflammatory cell infiltration after OECs transplantation. Therefore, more basic research is needed to fully clarify the immunomodulatory and anti-inflammatory characteristics of OECs, and only on the basis of a full understanding of this mechanism can be better over-applied to clinical trials. promote nerve injury repair and functional recovery by improving or enhancing the immune characteristics and anti-inflammatory ability of OECs.

## 8 Conclusion and prospect

Both basic and clinical trials have shown the advantages of OECs in the repair of nerve injury, but the mechanism of OECs in promoting nerve regeneration and nerve repair is still unclear. However, the immunogenicity and immunomodulatory function of OECs have been understood. First. OECs express a variety of cytokines, which can produce a variety of nutritional factors, growth factors and anti-inflammatory factors through paracrine, and can inhibit or reduce the expression level of pro-inflammatory cytokines and inhibitory factors, which provides a favorable microenvironment for nerves. Secondly, OECs can promote the transformation of immune cells to anti-inflammatory phenotype by regulating the activity and infiltration of immune cells, including macrophages, microglia and astrocytes, and by changing the polarization of these cells. reduce secondary tissue injury and nerve degeneration, protect neuron survival and promote axonal regeneration. Finally, OECs can guide the long-distance extension of newborn axons and bridge distal damaged nerves, provide strong support for immune function.

The application of OECs in the field of basic research in nerve injury treatment has achieved exciting and encouraging results, and the possibility of gradually transitioning to clinical trial applications. Although these current studies have revealed that OECs are feasible and safe in clinical trials, their therapeutic effects are not ideal and need to be verified by more data. This may be related to the existence of some key issues that need to be solved urgently. The origin of OECs is the first problem to be solved. The imperfect *in vitro* culture system, including the culture cycle, cell vitality and quality, and the possible potential factors that may affect the stability of OECs genes during the culture process, can lead to limited widespread access and application of OECs. Therefore, improving the *in vitro* culture system and obtaining a sustainable source of cells are the first problems to be solved, which may allow obtaining a sustainable source of OECs through somatic cell gene reprogramming technology to provide new hope for nerve repair. Secondly, significant progress has been made in the therapeutic application of OECs in animal models of nerve injury, but the therapeutic effect in clinical trials is controversial, which may be related to differences between species. Therefore, results obtained from basic research cannot be directly applied to clinical trials. More basic research is needed to understand the working mechanism of OECs in the body, fully grasp their nerve regeneration, anti-inflammatory and immunoregulatory functions to achieve effective therapeutic results. Thirdly, issues such as OECs transplantation method, transplantation time, transplantation cell dose, and transplantation cell localization and tracking *in vivo* are also important factors leading to differences in treatment effects. OECs can be transplanted into the host through a variety of methods (including venous transplantation, intrathecal transplantation, and local transplantation), but neither method can ensure whether the transplanted cells can colonize and survive the injury site for a long time. Transplanted cells may also have negative effects through blood circulation or penetration and metabolic problems in the body. There is currently no unified standard for the optimal time for cell transplantation, and it is unclear at which time point after nerve injury can be transplanted to achieve the best therapeutic effect. Whether higher doses of transplanted cells produce better therapeutic effects remains unclear. Therefore, these problems require more time and research to explore and solve them in the future. Although the application of OECs in the treatment of nerve injury is in the primary stage, the potential of OECs in the treatment of nerve injury cannot be denied. In short, OECs are a promising candidate for treatment.
